# Familial Russell–Silver Syndrome like Phenotype in the PCNA Domain of the *CDKN1C* Gene, a Further Case

**DOI:** 10.1155/2019/1398250

**Published:** 2019-12-22

**Authors:** A. H. Sabir, G. Ryan, Z. Mohammed, J. Kirk, N. Kiely, M. Thyagarajan, T. Cole

**Affiliations:** ^1^ Birmingham Women’s and Children’s Hospital NHS Trust and Birmingham Health Partners Birmingham, UK, nhs.uk; ^2^ Robert Jones & Agnes Hunt Orthopaedic Hospital Oswestry, UK, rjah.nhs.uk

## Abstract

We present two half siblings with significant short stature who proved a diagnostic challenge for several years. Radiological findings included subtle epiphyseal changes. The diagnosis was made through whole genome sequencing via the 100,000 genome project. A maternally inherited pathogenic heterozygous *CDKN1C* variant was found in the PCNA (proliferating cell nuclear antigen) domain. Mutations of the PCNA domain of the *CDKN1C* gene are known to be associated with IMAGe syndrome thus with adrenal disease, although neither affected patient in our case had evidence of adrenal dysfunction. This report supports the previously reported findings of Russell–Silver syndrome (RSS) like phenotype caused by this unusual mechanism (CDKN1C mutations in the PCNA domain), highlights subtle radiological features not described previously and the phenotypic variability between two affected siblings. Additionally it reminds clinicians of the importance of considering associated adrenal disease/diabetes mellitus for variants within the PCNA domain. Finally it confirms RSS‐like disorders should be considered in patients who have epiphyseal or metaphyseal changes and short stature, since CDKN1C PCNA domain mutations can result in this phenotype.

## 1. Introduction

Advances in genomic testing have led to new diagnoses in many patients who were previously without diagnoses. In some patients genome sequencing has resulted in reversal of previous diagnostic labels, as is reported in this case, where we discuss two half siblings with significant proportionate short stature, born of unrelated normal stature Caucasian parents. Initial radiological findings included subtle epiphyseal changes leading to a diagnosis of M.E.D (Multiple Epiphyseal Dysplasia). Subsequent whole genome sequencing via the 100,000 genome project identified a maternally inherited pathogenic heterozygous *CDKN1C* variant (c.836G>T; (p.Arg279Leu)) in the PCNA (proliferating cell nuclear antigen) domain, leading to a diagnosis of *CDKN1C*‐related RSS.

The identified variant has been previously reported and associated with familial RSS‐like phenotype in one large French family [1]. Kerns et al. have reported a nearby variant (*CDKN1C* c.843G>T p.Arg281Leu) in a large Ecuadorian family with familial RSS‐like phenotype though with additional early adult onset diabetes [2].

We discuss this unusual mechanism of RSS and the subtle phenotypic difference (as compared to more typical RSS) and how this impacts management.

## 2. Case

Patient 1 (A1) is a 15 year old Caucasian girl, the only child of unrelated parents. A1 presented with significant proportionate short stature (height <0.4^th^ centile, weight 9−25^th^ centile). She had four paternal half‐sisters of normal height and one maternal older half‐brother (patient 2, B1) with significant short stature (152 cm at age 21 years). Both parents were of normal stature. Her mother’s height was 161 cm (25–50^th^ centile) and she achieved menarche aged 12 years.

A1 was born small for gestational age (SGA) (birth weight 2.13 kg, <10^th^ centile) at 39^+3^/40 gestation by ventouse delivery for fetal distress. There were no concerns around her early developmental milestones or feeding, although she was short and her height remained around the 3^rd^ centile.

She suffered with recurrent otitis media with effusions during childhood, requiring repeated myringotomies and grommets aged 5 years resulting in tympanic membrane scarring and bilateral conductive hearing loss diagnosed aged 14 years for which she wears hearing aids.

Aged 12 years she suffered with daily retro‐orbital and temporal headaches, sometimes associated with blurred vision, which did not improve with *β*‐blockers (propranolol). An MRI scan of the head showed borderline cerebellar tonsillar ectopia, but considered too mild to be classed as a Chiari malformation. Her ophthalmological review (including fundus examination) was normal but she required glasses for hypermetropia aged 3 years and by age 12 years her prescription was +6.0 Dioptres, although there were only minimal changes in the prescription around the time of the headaches.

A1 was reviewed for short stature for the first time aged 12 years by the Paediatric Endocrinology team. The family did not present earlier due the lack of a diagnosis in her maternal half‐sibling (B1) who had a similar presentation of short stature and was fully investigated without achieving a diagnosis. The family and A1 also declined growth hormone (GH) due to previous poor response of GH in B1. By 12 she was in established puberty (breast stage 2‐3, pubic hair stage 1‐2) with menarche commencing age 12−13.

Aged 14 years, A1 began to have recurrent nosebleeds but these resolved with oral antihistamines and topical bactroban ointment. Her rhinosocopy was normal.

On examination she had a pointed chin (see Figure 1(a)) with small ears and a high anterior hairline. Her forehead was prominent (also present in her mother) as seen in Figures 1(a)and1(b). There was mild mid‐facial crowding with a slightly high arched palate. She had 5^th^ finger clinodactyly bilaterally, and incurving of the lateral aspect of her toenails bilaterally with superior curving of the anterior nail portions. There were significant proportionate short stature and joint laxity (Beighton score of 7/9). She had a <0.5 cm lower limb length discrepancy (the right leg shorter than the left). Her height at age 15 years was 143 cm (<0.4^th^ centile).

Figure 1(a and b) A1 aged 15 years. Facial profile demonstrating prominent forehead, high anterior hairline, and pointed chin.

**Figure:**
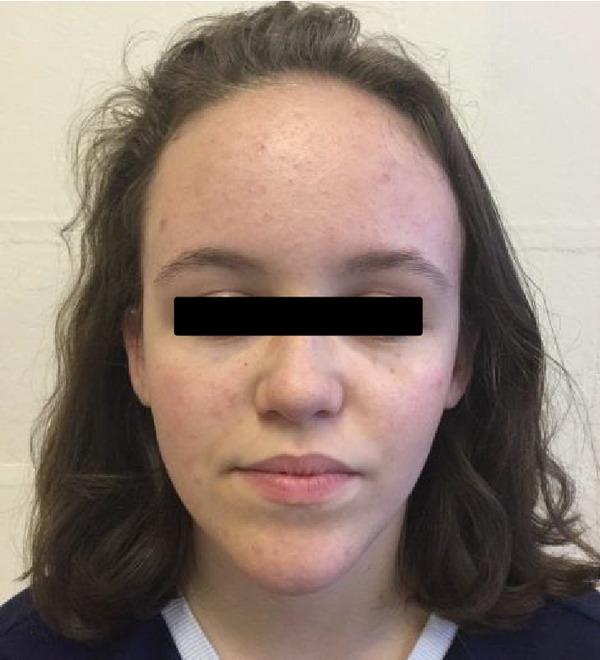
(a)

**Figure:**
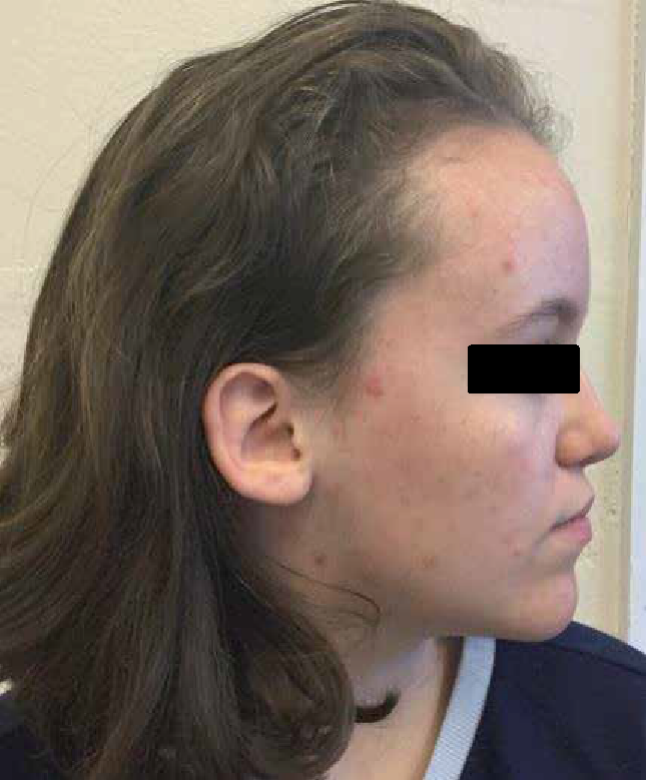
(b)

Figure 2(a and b) A1 with her maternal half‐brother, B1. Note significant short stature (both <0.4^th^ centile). A1 has lumbar lordosis.

**Figure:**
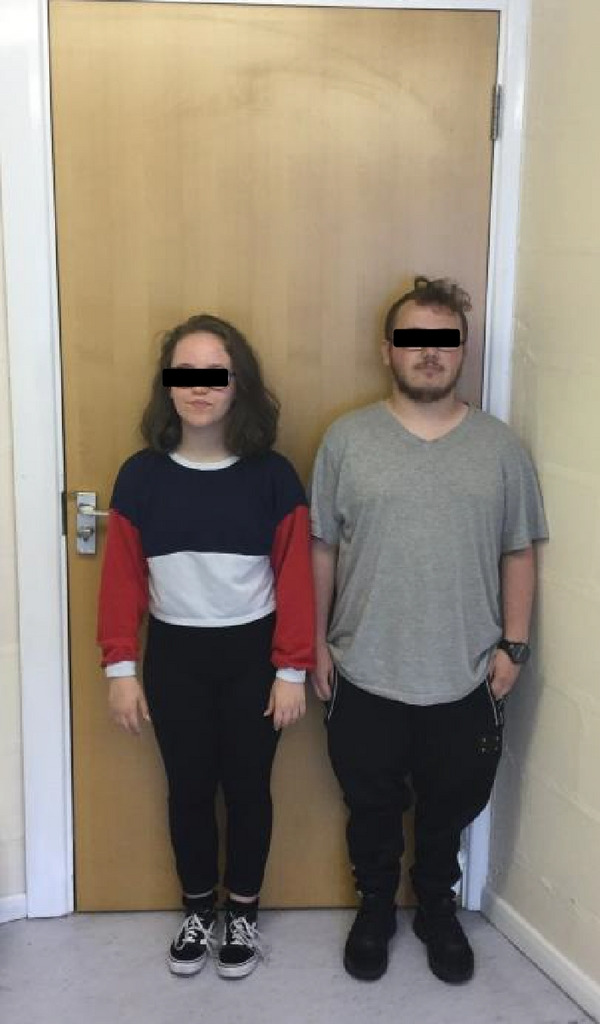
(a)

**Figure:**
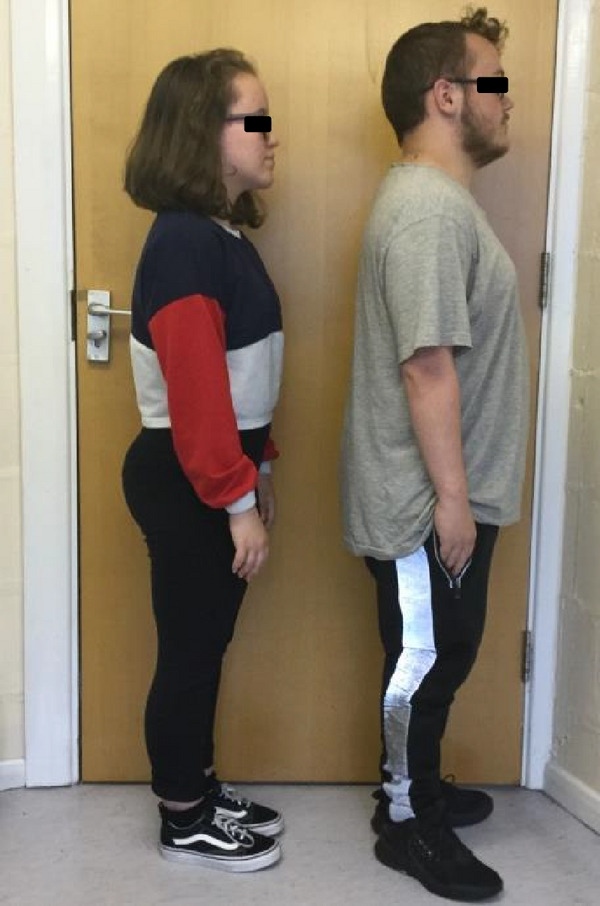
(b)

## 3. Family History

Her maternal half‐brother, B1, also presented with short stature. He was born at 41/40 weeks gestation via emergency Caesarean section due to low fetal heart rate, and was SGA with a birth weight of 2.07 kg (<0.4^th^ centile). He required some support via tube feeding in the first week of life, but was discharged breastfed on day 7. His height throughout childhood remained <0.4^th^ centile. He was treated with GH aged 5−8 years under the SGA license but this was stopped due to a poor response. The mother described him as a picky eater initially but he had no feeding issues after childhood.

There were no concerns with his early developmental milestones. He had some delay in tooth eruption and a double layer of front teeth for a while. During childhood he had asthma requiring occasional admissions and oral prednisolone courses, although he grew out of this by age 5‐6 years. Aged 5 years there were concerns regarding poor concentration, challenging behaviour and he was diagnosed as having dyspraxia aged 11 years. He found secondary school challenging and was diagnosed with Asperger’s aged 15 years. He went through puberty normally (age 11 years at onset with pubic hair growth, testicular, and scrotal growth) and his final height was 152 cm (<0.4^th^ centile). He is taller than A1 as shown in Figures 2(a) and 2(b) Human GH was administered from age 14 to 17 years under the SGA license (35 *μ*g/kg daily) but with poor response.

On examination he had a prominent forehead (although less than A1) (as seen in Figures 3(a) and 3(b), high anterior hairline, small hands, and feet, but nil else of particular note. He was also recruited into the 100,000 genome project.

Figure 3(a and b) B1 (aged 21 years) facial front view and profile. Note prominent forehead and high anterior hairline.

**Figure:**
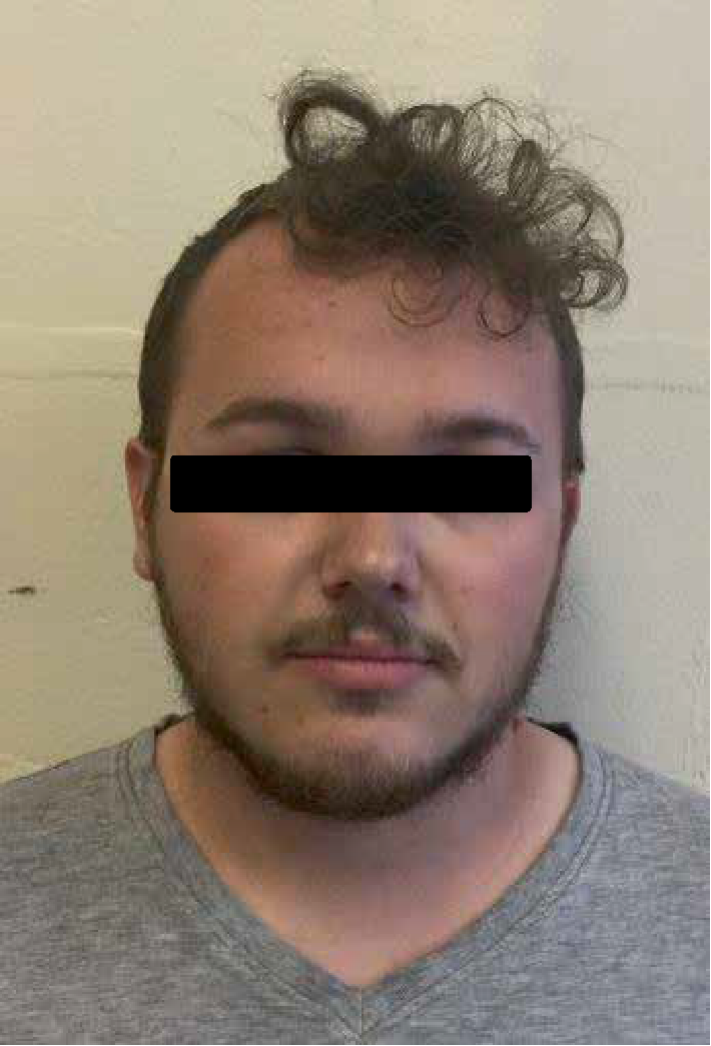
(a)

**Figure:**
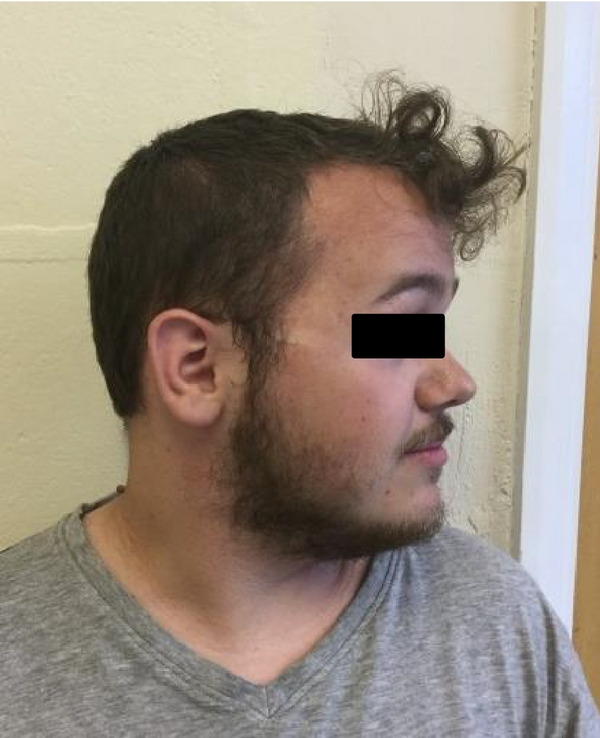
(b)

## 4. Investigations

### 4.1. Biochemistry

Aged 12 years A1’s; Calcium, Adjusted Calcium, Phosphate, Magnesium, and Alkaline phosphatase levels were all normal. Her FSH (8.1 U/L), LH (11.4 U/L), TSH (1.57 mU/L ref: 0.60−4.80), fT4 (16.6 pmol/L ref: 10.7−21.8), and Oestradiol (179 pmol/L) levels were normal. Her Insulin like growth factor (IGF‐1) was slightly raised above the normal at 68.7 nmol/L (11.4−51.9), indicating a degree of IGF‐1 resistance. Her random cortisol level was 197 nmol/L which is not suggestive of adrenal insufficiency but is only partially informative as it was taken at 12:00 hours rather than 09:00 hours. A follow‐up short synacthen test is awaited.

Aged 3 years B1’s; ft4 (15.7 pmol/L ref: 13.8−22.5), IGF‐1 (6.3 ref: 4.1−26.0), and alkaline phosphatase levels were all normal. His 09:45 hours cortisol level was 162 nmol/L (normal). His GH stimulation test was normal (aged 4 years) with peak GH levels of 16.0 mU/L.

### 4.2. Radiographs

The skeletal survey performed aged 13 years showed subjective hypoplasia of the distal radial epiphyses (Figures 4(a) and 4(b)). There was a slight lack of interpedicular widening of the vertebrae but no elongation of the fibula. The 4^th^ and 5^th^ metacarpals were bilaterally short (Figure 4(a)). There was no evidence of metaphyseal dysplasia. The bone age matched her chronological age at 12 years.

Figure 4(a and b) Radiographs of A1’s left hand and left forearm with changes described in the text.

**Figure:**
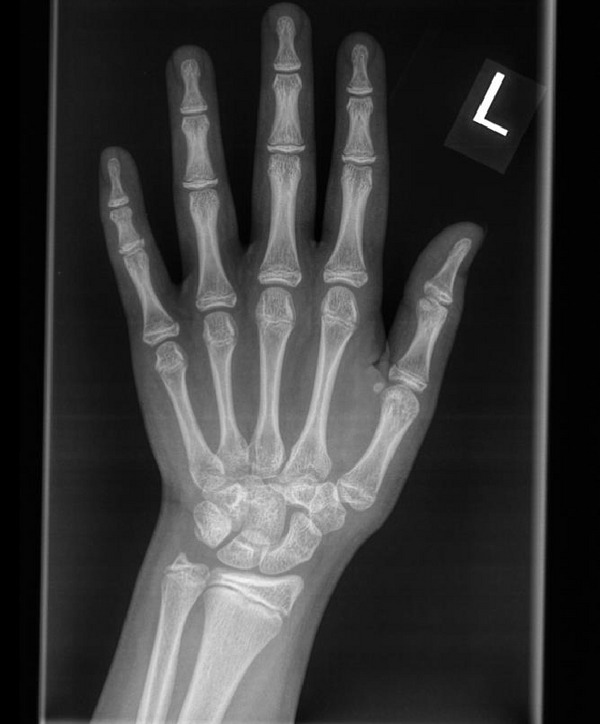
(a)

**Figure:**
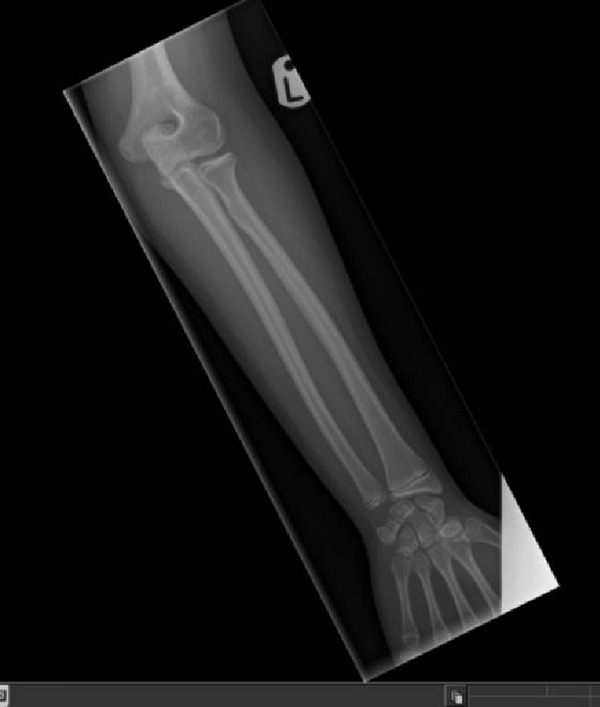
(b)

**Figure 6:**
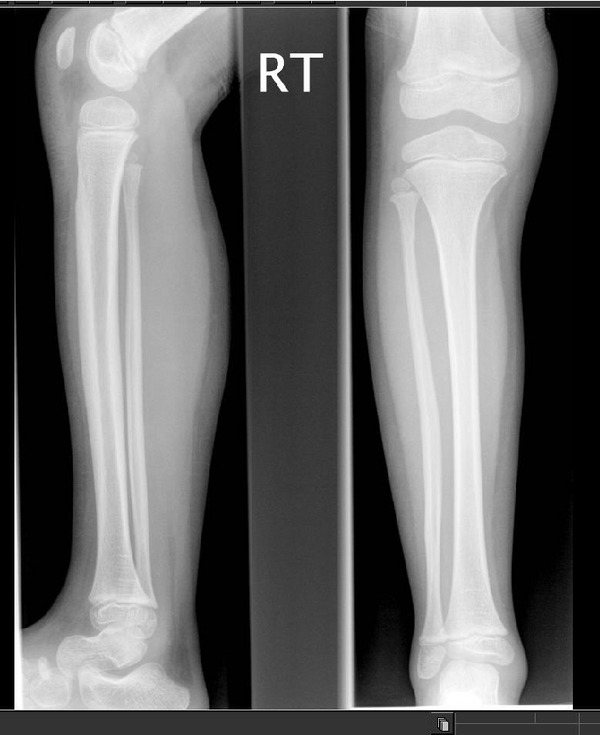
B1 radiograph of right lower limb (lateral and AP) aged 5 years.

B1’s radiographs (hand and lower limb images) showed slight global bone age delay (age 6 years) and subjective smaller epiphyses (Figures 5(a) and 5(b)), with slight elongation of the fibula (Figure 6). There was no evidence of metaphyseal dysplasia.

Figure 5(a and b) B1 radiograph of right hand aged 15 years (a). B1 radiograph of right hand aged 3 years (b).

**Figure:**
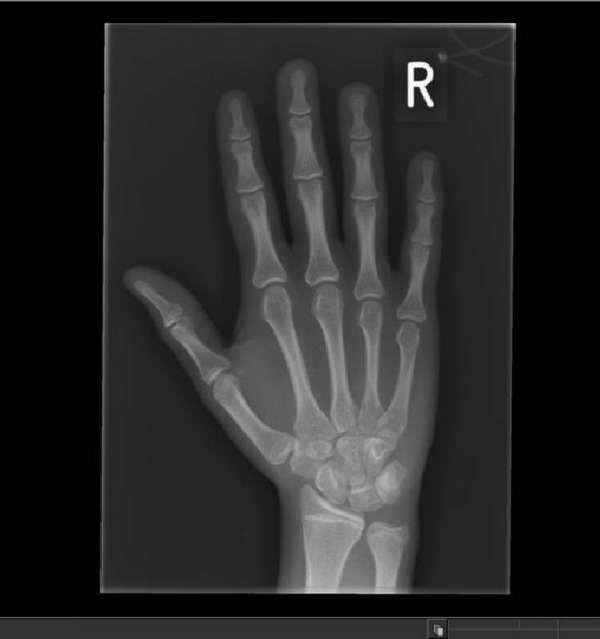
(a)

**Figure:**
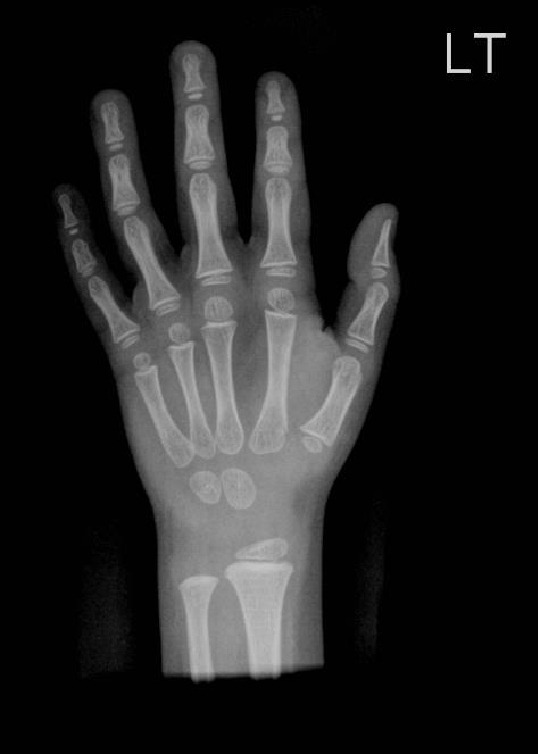
(b)

### 4.3. Initial Diagnosis

A1 was initially felt to have MED (multiple epiphyseal dysplasia) based on the subtle and variable epiphyseal changes on her radiographs. There were, however, some features that were atypical for MED including the low birth weight and severity of the short stature.

The MED diagnosis was questioned on further review aged 15 years, as the radiographic findings were subtle, and the severity of the short stature/subtle dysmorphology was out of keeping with MED. A diagnosis of MED also, made it difficult to explain the findings in her maternal half‐brother (as gonadal mosaicism would need to be the mechanism to cause MED in two offspring of an unaffected mother).

## 5. Genetic Testing

A1 had a normal Array CGH in 2016 (BlueGnome 8x60k v2.0 ISCA platform and analysed in BlueFuse Multi v4.1) and normal female karyotype, 46XX, with no copy number imbalances. Genetic testing for MED had not been initiated specifically as A1 had already been recruited to the 100,000 genome project.

Whole genome sequencing was performed as part of the 100,000 Genome Project Rare Disease arm, with the following panels applied based on A1’s phenotype: Unexplained skeletal dysplasia v1.104, congenital hearing impairment (profound/severe) v1.41, Multiple epiphyseal dysplasia v1.2, IUGR, and IGF abnormalities v1.24 [3].

**Figure 7:**
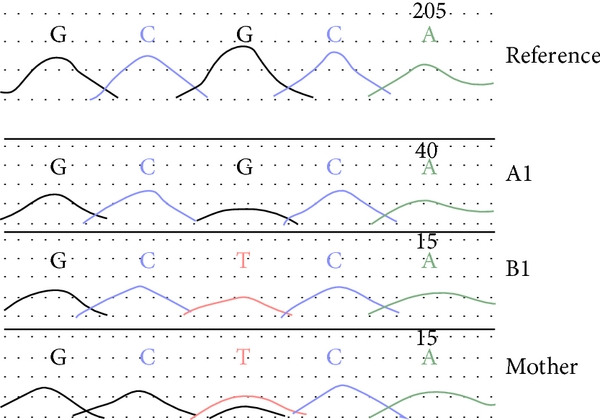
Sanger sequence analysis for A1, B1, and their mother showing nucleotide change C>A, heterozygous.

**Figure 8:**
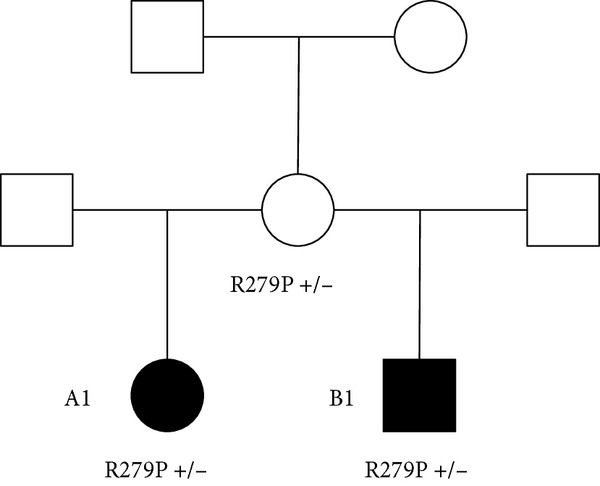
Focussed pedigree showing CDKN1C gene change; c.836G>T; p.(Arg279Leu) in A1, B1, and their mother.

A heterozygous maternally inherited variant in the *CDKN1C* gene was identified; c.836G>T; p.(Arg279Leu) (NM_000076.2) as shown in Figure 7. This was confirmed by Sanger sequencing in the local diagnostic lab (West Midlands Regional Genetics Laboratory) and confirmed in her mother and B1 (as illustrated in Figure 8).

### 5.1. Evidence

A1’s variant (CDKN1C, c.836G>T; p.(Arg279Leu)) is a heterozygous missense change. The *CDKN1C* gene is paternally imprinted and maternally expressed, and therefore is compatible with the segregation pattern in this family. We were unable to undertake genetic analysis in the generation prior to A1’s mother, though we know that these individuals had normal stature.

The amino acid Arginine at codon 279 is evolutionarily highly conserved, and multiple *in silico* tools predicted this change to be damaging to the protein (SIFT score; 0, PolyPhen‐2 score; 0.972). It is present within the PCNA‐binding domain of the CDKN1C protein, where pathogenic variants have previously been associated with IMAGe syndrome or with a RSS‐like phenotype. The exact function of this domain is not currently known, however a number of variants in this domain have previously been reported in the literature associated with a similar phenotype [4].

Missense variation within the *CDKN1C* gene is constrained over the entire gene (gnomAD *z* score = 1.93), however the domain where this variant is located appears to have reduced missense variation compared to other regions of the gene. Further, the variant is not present on the gnomAD population database, with high coverage of this region for both Exome and genome sequencing on gnomAD.

The c.836G>T; p.(Arg279Leu) variant has been reported once previously in the literature in several members of a family with a phenotype resembling Russell–Silver syndrome [1]. The variant segregated with the disease in all affected members of the family who were genetically tested, and there were several other female members of the family who had clinical features but were not tested. The affected individuals did not have adrenal insufficiency. A1 does not have random cortisol levels suggestive of adrenal insufficiency, although further investigation with a short synacthen test is being pursued.

*In vitro* functional studies performed by Brioude et al. demonstrated increased stability (gain of function) of the CDKN1C protein; this is not necessarily the physiological mechanism causing disease but does fit with the protein being a cell‐cycle inhibitor and increased stability causing growth restriction. A variant at the same codon, leading to a separate amino acid change (c.836G>C; p.(Arg279Pro)), has also previously been reported as causing disease in a patient with IMAGe syndrome [5].

Brioude et al. describes how variants in the PCNA domain of the *CDKN1C* gene causing IMAGe syndrome caused increased stability, more so than the variant found in A1, which might explain the additional adrenal disease seen in IMAGe syndrome patients.

The c.836G>T; p.(Arg279Leu) variant was analysed using ACMG and ACGS variant interpretation guidelines and has been classified as pathogenic by clinical scientists at the West Midlands genetics laboratory [6, 7]. Evidence used is shown in Table 1.

**Table 1:** Evidence criteria used for classification and strength level of evidence utilised.

Evidence criteria for pathogenicity	Strength level of evidence used	ACMG Code
Cosegregation with disease in multiple affected family members in a gene definitively known to cause disease (*n* = (1/2)^5^ = 1/32)	Strong	PP1
Located in a mutational hot spot and/or critical and well‐established functional domain without benign variation	Moderate	PM1
Absent from controls	Moderate	PM2
Missense change at an amino acid residue where a different missense change determined to be pathogenic has been seen before	Moderate	PM5
Multiple lines of computational evidence support a deleterious effect on the gene or gene product	Supporting	PP3
Well‐established *in vitro* or *in vivo* functional studies supportive of a damaging effect on the gene or gene product	Supporting	PS3
The prevalence of the variant in affected individuals is significantly increased compared with the prevalence in controls	Supporting	PS4
Patient’s phenotype or FH highly specific for gene	Supporting	PP4

## 6. Discussion

### 6.1. CDKN1C

CDKN1C codes for the CDKN1C protein (cyclin‐dependent kinase inhibitor) which is a negative regulator of cell proliferation and is paternally imprinted (thus maternally expressed). Mutations in the gene are associated with BWS), IMAGe syndrome, atypical IMAGe, and RSS. BWS is a molecular and clinical mirror to RSS.

Missense mutations within a specific 10 residue amino‐acid sequence in the PCNA binding domain are associated with IMAGe syndrome and one familial report of RSS [1, 2].

### 6.2. Russell–Silver Syndrome

Russell–Silver syndrome (RSS) (OMIM~#80860) is a growth disorder characterised by the features in Table 2; the Netchine–Harbison clinical scoring system (NH‐CSS, 2015), which is 98% sensitive for detecting RSS patients with demonstrated molecular abnormalities. It no longer mandates SGA as a mandatory diagnostic feature. Individuals who meet 4 out of 6 criteria are considered to have RSS [8].

**Table 2:** Netchine–Harbison clinical scoring system (NH‐CSS) for RSS (2015).

Clinical features	Detail
(1) Prenatal growth retardation	Birth length and/or weight ≤−2SDS
(2) Post natal growth retardation	Height ≤−2SDS
(3) Relative macrocephaly at birth	Head circumference at birth at least 1.5 SDS above birth weight and/or length
(4) Protruding forehead	In toddlers (age 1–3 years)
(5) Body asymmetry	Defined as a leg length discrepancy (LLD) of ≥0.5 cm or arm asymmetry or LLD <0.5 cm with at least two other asymmetrical body parts (with one being a non‐face part)
(6) Feeding difficulties/BMI < 2 SDS	In toddlers

Minor features include 5^th^ finger clinodactyly, triangular facies, forehead prominence, and a short arm span. Supportive features include café‐au lait spots, genitourinary anomalies, developmental delay, and hypoglycaemia [1].

RSS is caused by defects to the genes on either chromosome 7 or 11. The commonest cause (in ~55% of patients) is a methylation defect at the 11p15 locus; loss of the IC1 domain methylation on the paternal allele, followed by maternal uniparental disomy of chromosome 7 (UPD7) in 7−10% of cases and without an identifiable cause in about ~35% of cases [1]. Other rare mechanisms include sequence changes in the ICR1 domain or the CDKN1C gene. Common causes of RSS (methylation defects and UPD7) are often sporadic events with low recurrence risk, whereas the aforementioned rarer sequence mechanisms can transmit in an autosomal dominant fashion.

Brioude et al. investigated a large cohort of patients with RSS without molecular cause published in 2013 and described a novel CDKN1C mutation (c.836G>[G:T} p.Arg279Leu) in a large French family causing RSS‐like phenotype (IUGR, relative macrocephaly, short stature with normal adrenal function) with no evidence of IMAGe. All individuals were female, so testicular volume could not be assessed. All the affected patients inherited the variant maternally (consistent with paternal imprinting of CDKN1C, the mutated gene is not expressed/is silenced when paternally inherited), thus completing the molecular mirror with BWS inheritance [1].

LoF (loss of function) variants of CDKN1C increase growth (remove the growth suppression brake), whereas GoF (gain of function) CDKN1C variants lead to further growth suppression. Functional studies by Brioude et al. showed increased stability of the CDKN1C protein with the c.836G>T; p.(Arg279Leu) variant. RSS shares several of its features (pre and post natal growth retardation, prominent forehead) with a very rare condition called IMAGe syndrome.

### 6.3. IMAGe Syndrome

IMAGe syndrome is a rare condition characterised by severe IUGR (I) in addition to postnatal growth restriction resulting in proportional short stature, metaphyseal dysplasia (M), congenital adrenal hypoplasia (A), and genital anomalies (Ge) in males [OMIM ~ #614732]. Skeletal abnormalities in IMAGe most commonly are delayed bone age and short stature but, occasionally, metaphyseal, and epiphyseal dysplasia of varying severity. Adrenal insufficiency often presents in the first month of life as an adrenal crisis or rarely later in childhood with failure to thrive and recurrent vomiting. Genital abnormalities present in males (as cryptorchidism, micropenis, and hypospadias) but not in females. Hypotonia and developmental delay are reported in some patients and cognitive outcome appears to be normal in the majority [9]. At least six gain of function mutations in the *CDKN1C* gene have been found to cause the condition [10].

Kerns et al. reported (in JCEM in 2014) a novel mutation causing a new disorder associated with features of the IMAGe syndrome, but without adrenal insufficiency or metaphyseal dysplasia (based on a large Ecuadorian family of 15 affected members over 6 generations). The phenotype observed in the family was less severe than that seen in IMAGe syndrome and included IUGR, failure of an adolescent growth spurt, proportional short stature, minimal subluxation of the 5^th^ metacarpal‐phalangeal joint, and early adulthood‐onset diabetes, unrelated to obesity or other manifestations of metabolic syndrome. Transmission to a child with a resultant phenotype only occurred following maternal inheritance, suggesting the disorder was paternally imprinted. The Kerns paper identified the *CDKN1C* c.843G>T p.Arg281Leu heterozygous missense variant through exome sequencing of 5 affected individuals.

Kerns reported that in their group, characteristic features of the RSS such as the body asymmetry, frontal bossing, café au lait spots, downturned corners of the mouth, and triangular face shape were rare. The features of RSS specific to the affected individuals were IUGR and severe short stature (proportional). There was no evidence of reproductive difficulty in their population. ACTH levels were normal, stimulation testing had not been performed and the adrenal glands were smaller than usual though this could have been due to smaller body size. Of the 15 affected, 8 had diabetes before age 40 years, and 2 unaffected individuals were still under 20 years.

## 7. Conclusion

The identification of a pathogenic CDKN1C variant in A1 and B1, matches the phenotype of short stature, SGA, characteristic facies without apparent adrenal insufficiency, and subtle epiphyseal changes in A1, and severe short stature, and feeding difficulties in B1. A1’s mother had normal stature with no features in keeping with RSS/IMAGe syndrome, although she did carry the CDKN1C variant. This suggests that she inherited the variant from her father (which is consistent with the inheritance of this condition), though we were unable to test this.

This presentation of familial RSS is similar to that described by the large French family by Brioude et al. with a lack of diabetes mellitus, whereas several members of the Ecuadorian family reported by Kerns et al. had early adult‐onset diabetes. We note that both our patients are still young (15 years and 21 years respectively) so need monitoring for the possible development of diabetes. Our case highlights differences with the large French family with familial RSS (Brioude) with the same variant (c.836G>T; p.(Arg279Leu)) in that A1 had some mild epiphyseal changes. This is important to consider as this initial skeletal finding indicated MED rather than RSS, but this family emphasises that subtle epiphyseal changes should not exclude a diagnosis of atypical RSS. Epiphyseal changes (as well as metaphyseal changes) have been described in the IMAGe syndrome [6].

CDKN1C is already known to cause BWS (LoF variants), IMAGe syndrome (PCNA domain GoF variants), and an atypical version of RSS. The atypical version of RSS is caused by mutations in the PCNA domain where mutations usually cause IMAGe syndrome. The CDKN1C related atypical RSS though within the mutational hotspot region of the IMAGe syndrome it does not exhibit adrenal insufficiency nor metaphyseal dysplasia as reported in the literature thus far.

The PCNA‐related RSS has a lack of some of the usual features of RSS, but also has some additional features. Additional features include early‐onset of adulthood diabetes (seen with the c.843G>T p.Arg281Leu variant). The cause for diabetes has been associated with increased expression of CDKN1C in the pancreatic B cells. The lack of usual features includes rarity of body asymmetry, frontal bossing, café au lait spots, and downturned corners of the mouth [2].

Though no apparent adrenal insufficiency was observed in either the family in this study or those reported by Brioude or Kerns, we suggest that patients with RSS due to the CDKN1C PCNA domain‐related mutations should have a short synacthen test to rule it out. This is especially important in our case since mutations of the codon (279) have been reported in IMAGe as mentioned by Kerns.

## Conflicts of Interest

The authors declare that they have no conflicts of interest.
